# [Retracted] Knockdown of TRIM9 attenuates irinotecan‑induced intestinal mucositis in IEC‑6 cells by regulating DUSP6 expression via the P38 pathway

**DOI:** 10.3892/mmr.2025.13504

**Published:** 2025-03-27

**Authors:** Wenjun Zhao, Qingming Wang

Mol Med Rep 24: 867, 2021; DOI: 10.3892/mmr.2021.12507

Following the publication of this paper, it was drawn to the Editors’ attention by a concerned reader that certain of the β-actin control data shown in the western blots in Fig. 3E on p. 6 were strikingly similar to data appearing in different form in other articles written by different authors at different research institutes that had already been published elsewhere prior to the submission of this paper to *Molecular Medicine Reports*. Owing to the fact that the abovementioned data had already apparently been published previously, the Editor of *Molecular Medicine Reports* has decided that this paper should be retracted from the Journal. The authors were asked for an explanation to account for these concerns, but the Editorial Office did not receive a satisfactory reply. The Editor apologizes to the readership for any inconvenience caused.

